# Psychological constructs and preferences for a complementary inclusive health insurance: a hybrid choice model

**DOI:** 10.1093/heapol/czaf056

**Published:** 2025-08-21

**Authors:** Qun Wang, Jinnan Wang, Shuwei Zhang, Fengyun Yu, Manuela De Allegri

**Affiliations:** School of Public Administration and Policy, Dalian University of Technology, Linggong Road 2, Dalian 116024, P.R. China; School of Public Administration and Policy, Dalian University of Technology, Linggong Road 2, Dalian 116024, P.R. China; Center for Chinese Public Administration Research and School of Government, Sun Yat-sen University, No. 132, Waihuan East Road, Guangzhou City, Guangdong Province 510006, P.R. China; Interdisciplinary Centre for Scientific Computing, Heidelberg University, Im Neuenheimer Feld 205, 69120 Heidelberg, Germany; Heidelberg Institute of Global Health, Heidelberg University Hospital and Faculty of Medicine, Heidelberg University, Im Neuenheimer Feld 130.3, 69120 Heidelberg, Germany

**Keywords:** psychological constructs, preferences, complementary inclusive health insurance, hybrid choice analysis, discrete choice experiment

## Abstract

Many low- and middle-income countries are affected by catastrophic health expenditures due to overstretched public health financing, indicating need for complementary inclusive health insurance solutions. Promoting these solutions requires understanding drivers, including psychological determinants, of health insurance purchase. Yet, relevant evidence is lacking. We employed hybrid choice models to analyze discrete choice experiment data and examine preferences for Huimin Insurance, a widely diffused complementary inclusive health insurance in China. We relied on KuRunData to collect our discrete choice experiment data. We found that people who regarded themselves to be at greater health risk preferred more generous benefits, while they did not value strong government involvement in product operation, design, and publicity. Higher scheme awareness was associated with a greater propensity to purchase coverage, to be willing to pay higher premium, and to accept lower reimbursement rates. High awareness coupled with a low perception of the scheme value resulted in a preference for an expanded package covering prevention and screening services. Stronger value was attributed to the Huimin Insurance among population groups that lack access to other insurance products, such as women and rural residents. By integrating psychological constructs in the decision-making analysis, we provide new evidence to guide design and promotion of appropriate health insurance schemes, especially catastrophic diseases.

Key messages

- Prior research has generally not considered the role of psychological factors when examining preferences for health insurance. We relied on a discrete choice experiment and hybrid choice models to consider also psychological factors in our analysis of preferences for Huimin Insurance, a complementary inclusive health insurance in China.
- Our analysis revealed that preferences for Huimin Insurance were shaped by individual perceptions of one’s own health risks, individual perceptions of the value of health insurance, and actual product knowledge.
- The evidence generated by our study is more comprehensive than what is currently available and as such, essential to inform the design of adequate policies to expand coverage and financial protection in China and other middle-income countries facing challenges similar to China.

## Introduction

The world is far from reaching its 2030 Sustainable Development Goal 3.8, i.e. achieving universal health coverage. The proportion of the population suffering from catastrophic out-of-pocket health expenditure has increased from 9.6% in 2000 to 13.5% in 2019 (World Health Organization 2023). Many low- and middle-income countries (LMICs) still rely on out-of-pocket expenditure as one of the major sources of healthcare spending (Hooley *et al.* 2022). Considering that most LMICs are less likely to have enjoy sufficient social health insurance coverage to finance access to health services, more and more countries are incorporating complementary private health insurance solutions to provide coverage for services excluded or only partially covered by social health insurance (Brunner *et al.* 2012, Zhang *et al.* 2020). In China, due to the existence of ceilings, limited reimbursement rates, and the narrow coverage scope of the country’s basic social health insurance scheme, catastrophic health expenditure is still one of the leading factors causing ill people to fall into the poverty trap (Xu and Huang 2023). Due to an increasing aging population, basic social health insurance funds are slowly depleting in many parts of China (Wu *et al.* 2023), challenging the much needed expansion of benefit packages. Traditional private health insurance plays an insignificant role in China’s health financing system due to its high premiums and strict restrictions on pre-existing conditions (Yu *et al.* 2021). Against this background, in recent years, the Chinese government has issued several policies to support commercial insurance companies to develop inclusive health insurance products that can provide complementary protection for catastrophic illness events (The Central Committee of the Communist Party of China and the State Council of China 2020, National Financial Regulatory Administration 2024). Such support stems from the country's vigorous efforts to develop complementary social security, especially in the areas of health insurance, pensions, and long-term care (National Development and Reform Commission 2021), which mainly rely on market mechanisms and the non-governmental sector for implementation (He 2023).

In this context, many commercial insurance companies have successively launched Huimin Insurance, also known as a complementary inclusive health insurance, in various parts of China. Huimin Insurance provides commercial coverage for catastrophic health spending. Schemes are usually characterized by government involvement, low premiums, no restrictions on pre-existing conditions, and high deductibles (Shen *et al.* 2023). The practice of Huimin Insurance has formed a new collaboration model between government and market—the government provides guidance in design, facilitating promotions, data support, supervision, etc. (i.e. using the government's reputation to “endorse” Huimin Insurance), guiding commercial insurance companies to provide inclusive catastrophic disease protection for its people; the public participates in the construction of complementary inclusive health insurance by voluntarily paying premiums. The development of Huimin Insurance is of great significance to the construction of China's multi-level social security system. At present, 23 provinces and 129 cities have launched Huimin Insurance products. In the field of long-term care, some areas have drawn on the experience of Huimin Insurance and launched complementary inclusive long-term care insurance (Xu 2024). However, as a voluntary private insurance, most Huimin Insurance products have low enrollment rates, and the scheme is therefore far from achieving its goal of becoming a real complementary insurance. In 2023, although the number of people covered by Huimin Insurance nationwide reached 160 million, 74% of such products had an enrollment rate of less than 15% (Xu 2024).

Increasing enrollment in Huimin Insurance is not a straightforward endeavor, considering the specific characteristics of the scheme. First, Huimin Insurance provides protection for “low probability, high loss” catastrophic disease events, which people often underestimate, either because they are overly optimistic or short-sighted (Kunreuther 1978). Such misunderstanding of catastrophic disease risk can easily lead people to reach the “irrational” decision not to purchase insurance coverage. Second, the operation of Huimin Insurance involves multiple stakeholders with different, and at times contrasting, interests. One needs to understand that commercial insurance companies provide Huimin Insurance to pursue a financial gain, while local governments expect Huimin Insurance to result in greater benefits for their communities (Hu *et al.* 2023). The divergence between these two sets of interests poses challenges to the product design of Huimin Insurance.

Factors affecting health insurance enrollment have always been a topic of concern among researchers and policymakers. Most discussions on this topic have been based on expected utility theory, which assumes that individuals possess all appropriate information about health insurance and are able to make rational tradeoffs between costs and benefits of different schemes in order to maximize their utility when choosing whether to pay premium to purchase or not to purchase coverage (Mongin 1998). From this perspective, premiums and benefits are key insurance attributes affecting health insurance demand. Other researchers have further identified that people’s preferences for health insurance are influenced by their state, defined in terms of their socioeconomic, demographic, and health status (Schneider 2004). Due to its strict assumptions, expected utility theory has faced significant challenges in explaining demand for health insurance in practice. Behavioral economics combines psychology and economics and relaxes the strict assumptions of expected utility theory, including those of rational agents. As a result, behavioral economics is regarded as an effective tool to support the design health of health insurance plans and related interventions to expand insurance coverage (Baicker *et al.* 2012).

However, up to the time of submission, we could not identify any studies that have linked psychological constructs to people’s preferences for health insurance, in China nor elsewhere. This is surprising given that psychological constructs are often integrated into people’s preferences for other products or services under the framework of the hybrid choice model (HCM) (Alemu and Olsen 2019, Arora *et al.* 2022). The absence of such research in the context of health insurance suggests a gap in understanding how psychological factors influence individuals’ choices in this critical area. Many health insurance preference studies have used discrete choice experiments (DCEs) to simulate the decision-making process of health insurance purchase. In such studies, insurance attributes and socioeconomic and demographic factors are used as explanatory variables for choice behavior (Ozawa *et al.* 2016, Wang *et al.* 2021b). McFadden stated that such analyses usually neglect psychological factors in the process of making choices, leaving people’s cognitive drivers to be defined as “black boxes” (McFadden 2001). From the perspective of behavioral decision-making, choice itself is better explained when combining both “soft information” (psychological factors) and “hard information” (socioeconomic and demographic factors). Based on a HCM and a DCE, our study aimed to fill this knowledge gap by exploring the role of psychological constructs (such as risk perceptions and insurance perceptions), insurance attributes, and socioeconomic and demographic status in shaping preferences for Huimin Insurance.

## Conceptual framework

Based on the above literature review, we concluded that psychological constructs, insurance attributes, and socioeconomic and demographic factors are the three main groups of factors that affect people’s demand for health insurance at the micro level. This constituted the basis for our conceptual framework. We then used the following theories and studies to further formulate the conceptual framework. Prospect theory, as the basis of behavioral economics, describes the value of a prospect through weight and value functions. The weight function reflects the individual's subjective judgment on the probability of an event occurring. And the value function emphasizes the individual's subjective value on losses and gains of an event (Kahneman and Tversky 1979). Followed by Simon’s notion of “bounded rationality,” Kunreuther (1978) established the sequential model of insurance choice and viewed insurance purchase as a “decision-making process” rather than a “one-time event.” This process includes three sequential stages: in the first stage, individuals perceive risk as a problem; in the second stage, individuals are aware of insurance; in the third stage, individuals think insurance is valuable to purchase. Informed by the literature, we included perceptions of health risk, awareness of Huimin Insurance, and perceptions of its value as the key dimensions of psychological constructs. Considering the sequential nature of insurance choice, we further postulated that a higher health risk perception would be positively associated with awareness of Huimin Insurance and with a positive perception of its value, and that awareness of Huimin Insurance would be positively associated with perceptions of its value.

With regard to insurance attributes, we recognized the existence of differences in premiums and benefits (the dimensions identified based on expected utility theory) across various regional Huimin Insurance schemes. Another main difference lay in the degree of government involvement. Government involvement levels positively influenced enrollment rates. Several regional governments in China are either deeply or superficially involved in the product design, publicity, and operation of Huimin Insurance. Most governments are involved moderately in the operation processes of Huimin Insurance (China Re Life 2023). We thus added government involvement as another insurance attribute.

As for socioeconomic and demographic factors, we included two dimensions. One included general socioeconomic and demographic factors affecting health insurance demand. The other included specific factors targeted for Huimin Insurance, such as whether the respondent or any of his/her family members have ever suffered from a catastrophic illness event.

## Materials and methods

### Study setting and Huiliao Insurance

We conducted our study in Liaoning Province where Huiliao Insurance is on offer, a type of Humin Insurance based in Liaoning Province. Liaoning is a northeastern province in China with a medium level of economic development. All participants of social health insurance in Liaoning Province (excluding Dalian City) are eligible to participate in Huiliao Insurance. The scheme is jointly guided and supported by the Liaoning Province Health Security Bureau and the Liaoning Province Financial Supervision and Administration Bureau. More than ten commercial health insurance companies jointly launched Huiliao Insurance. It was first launched at the end of 2022, with a first-year participation rate of 5.06%. The 2023 Huiliao Insurance covered benefits within and outside the social health insurance catalog with a premium of 128 RMB and discounts on drug purchases as a value-added services.

### Discrete choice experiments development and experimental design

We used a DCE to capture people’s preferences. In line with our conceptual framework, we relied on the document analysis of about 140 Huimin Insurance plans (Wang *et al.* 2024) to identify nine possible attributes. Through a series of qualitative interviews with seven policymakers, six managers from insurance companies, three managers from third-party operation and promotion companies, and three academic researchers, we excluded the following attributes: (i) the participation of third-party companies—these companies directly served insurance companies and thus had little influence on people’s preferences for Huiliao Insurance; (ii) reimbursement ceiling—very few people have so far claimed up to the ceiling, which serves more as a publicity stunt. We therefore did not include it as an essential attribute; (iii) benefits for pre-existing conditions—those with pre-existing conditions have a higher willingness to participate, since they are usually excluded from traditional private health insurance. Nonetheless, healthy respondents were informed that individuals with pre-existing conditions would be allowed to enroll already during the DCE introduction.

Our final design included five attributes (Table 1). Attributes on benefit packages and deductibles were combined in a single attribute to simplify the respondents’ decision processes. Afterwards, considering that many Huimin Insurance schemes in other areas reduced their deductibles and increased their premiums and reimbursement rates in recent years, we selected the current levels of premiums, deductibles, and reimbursement rates of other more attractive plans as the improvement levels of the related attributes. And we used the current levels of premium, deductible, reimbursement rate, and value-added services of the 2023 Huiliao Insurance as the base levels. For the attributes of government involvement and value-added services, we also relied on the experiences of other Huimin Insurance plans. We illustrated the attribute of deductibles, the meaning of which some respondents might not understand, using a figure.

**Table 1.** Characteristics of attributes and attribute levels.

Attributes	Attribute levels	Specific definition	Anticipated utility form	Hypothesized signs
Individual premiums	130 RMB/year	Individual premium actually paid	linear	−
	170 RMB/year			
	210 RMB/year			
Government involvement	Purely commercially operated	Government basically did not involve in product operation, design, publicity, etc.	Non-linear	Reference
	Moderate government involvement	Government moderately involved in product operation, design, publicity, etc.		+
	Strong government involvement	Government deeply involved in product operation, design, publicity, etc.		+
Deductibles of benefits within and outside the social health insurance catalog	120 000 RMB	Deductibles of benefits within and outside the health insurance catalog were both at 120 000 RMB, or 150 000 RMB, or 180 000 RMB.	Linear	-
	150 000 RMB			
	180 000 RMB			
Reimbursement rates	50%	Ceiling levels considering a reimbursement coverage equivalent to 70% of total costs for items included in benefit package	Linear	+
	65%			
	80%			
Value-added services	Basic	Discounts on drug purchases	Non-linear	Reference
	Expanded 1	Same as basic + prevention and screening services (early screening for serious diseases, discounts on physical examinations, etc.)		+
	Expanded 2	Same as basic + disease diagnosis and treatment services (video consultation, traditional Chinese medicine services, etc.)		+
	Expanded 3	Same as basic + convenience in medical treatment (outpatient green pass, guidance in seeking care from experts, etc.)		+

To derive our scenarios, we applied a D-efficient design with the help of Ngene. We then organized a pilot DCE applying an online data collection platform, KuRunData, and using the same design as our final DCE. The sample size in the pilot was 60, with the same quota distribution as for the sample in the final survey. We excluded these 60 respondents from the final analysis sample. Based on the priors from the pilot, we finalized our DCE questionnaire. In the formal design, we had 24 choice sets, distributed in four blocks. Each choice set included three choices with two Huiliao Insurance packages and an opt-out. In addition, each respondent was presented with one extra choice set specifically designed as a quality control task, bringing the total number of choice sets per respondent to seven. The quality control choice set (Choice Set 7) was constructed to include one clearly most favorable and one clearly least favorable insurance option. This task was used to test internal consistency and assess the attentiveness of respondents. In both the pilot and the formal DCE, such a dominant alternative was included for this purpose. All respondents passed the internal consistency test in the pilot. The formal DCE questionnaire (Block 1) is shown in Supplementary Appendix 1.

### Data and data collection

KuRunData invited individuals from its sample database to participate in the online survey for this study in September 2023. KuRunData belongs to the Toluna Group and covers a sample of 9.5 million in China with 690 000 in the Northeast region (KuRunData 2023). KuRunData used quota sampling to recruit participants, thereby ensuring that the sample closely reflects the general population of Liaoning Province in terms of gender, age, education, area of residence, and city. In line with the area where Huiliao Insurance is offered, we collected data in all cities except Dalian City in Liaoning. Overall, 1203 respondents finished the whole questionnaire. Among them, 67 respondents selected the least favorable option in the quality control task, indicating potential inattention or misunderstanding, and were therefore excluded from the final analysis. This yielded a final valid sample of 1136 respondents.

### Psychological constructs and other variables

In line with prior research (Mathur *et al.* 2015; Abdilwohab *et al.* 2021), we developed scales to measure awareness of the Huiliao Insurance and perceptions of its value. The awareness indicators can be translated as: (i) “I know Huiliao Insurance,” (ii) “I think Huiliao Insurance is a complementary insurance to basic social health insurance schemes,” and (iii) “I think Huiliao Insurance is an inclusive private health insurance.” The indicators of perceptions of its value can be translated as: (i) “I think it is valuable to enroll in Huiliao Insurance,” (ii) “I think Huiliao Insurance can reduce the financial burden of catastrophic diseases,” and (iii) “I am satisfied with Huiliao Insurance.” For health risk perceptions, we adapted the classical dimensions of risk perceptions (Brewer *et al.* 2007) to catastrophic disease events, based on local applications (Guo *et al.* 2016). The indicators for health risk perceptions related to catastrophic disease included: (i) “It is difficult to keep your body healthy all the time and not suffer from catastrophic diseases,” (ii) “Risks to your health are everywhere,” (iii) “More and more people suffer from catastrophic diseases. I am very worried about my health,” (iv) “Due to environmental pollution, food safety issues, and increased life pressure, the possibility of suffering from catastrophic diseases is increasing,” and (v) “Once someone becomes seriously ill, it will bring unbearable medical expenses to the family.” All these psychological indicators utilized a five-point Likert Scale (“1 = Strongly Disagree”, “2 = Disagree,” “3 = Neutral/Uncertain,” “4 = Agree,” and “5 = Strongly Agree”). In the pilot, we also investigated the reliability and validity of our psychological scales. The Cronbach’s alpha coefficients for the latent variables of health risk perceptions, awareness of Huimin Insurance, and perceptions of its value were 0.790, 0.806, and 0.828, respectively. The confirmatory factor analysis (CFA) demonstrated that the composite reliability of the three scales were 0.790, 0.779, and 0.828, respectively, and the average variance extracted for the three scales were 0.435, 0.552, and 0.617, respectively.

In this study, we included age (classified as three groups: 18–34; 35–59; 60–75), gender, and area of residence as general socioeconomic and demographic factors, as well as having private health insurance and whether the respondents or any family members ever suffered from any catastrophic disease events as specific factors targeted by Huimin Insurance.

### Analytical model

**Figure 1.**
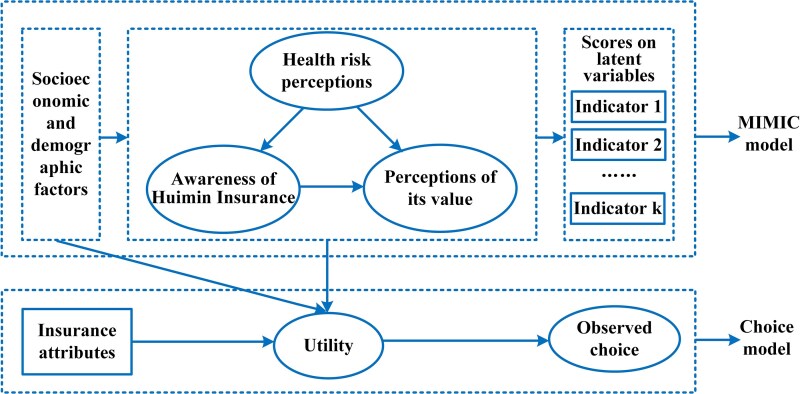
Framework of HCM.

We used a HCM for data analysis. HCM is an integration of a DCE and a latent variable model. We did not place psychological indicators directly into the choice model as observed variables since this may easily lead to measurement error (because psychological indicators are only the function of psychological constructs) and endogeneity bias (because there may exist unobservable variables that both effect psychological indicators and stated choices) (Ben-Akiva *et al.* 2002; Arora *et al.* 2022). Thus, we treated psychological indicators as latent variables in order to link them into a DCE (Fig. 1). The DCE and the latent variable model were estimated simultaneously in this study using the Apollo package for R. Supplementary Appendix 2 shows the latent variable model and the choice model of the HCM in this study.

## Results

### Descriptive statistics and data quality analysis

Out of the total 1136 respondents, 584 (51%) were men, 803 (71%) were from urban areas, and 531 (47%) had at some stage bought private health insurance. The average age was 46 years with the SD being 13 years. Among all the respondents, 193 (17%) reported that either themselves or their family members suffered from catastrophic disease events. The average annual per capita household income was 24 061 RMB, and 20.4% of the respondents had received college or above education. The sample distribution across different cities was as follows: Shenyang (217, 19.1%), Anshan (125, 11.0%), Fushun (75, 6.6%), Benxi (44, 3.9%), Dandong (77, 6.8%), Jinzhou (87, 7.7%), Yingkou (65, 5.7%), Fuxin (54, 4.8%), Liaoyang (58, 5.1%), Panjin (45, 4.0%), Tieling (101, 8.9%), Chaoyang (102, 9.0%), and Huludao (86, 7.6%). The vast majority of respondents always chose a Huiliao Insurance alternative with 22 (0.34%) choosing the opt-out. Among the total people surveyed, 434 (38%) reported being aware of Huiliao Insurance, 181 (16%) reported having no knowledge, while 521 (46%) held uncertain opinions. 547 (48%) agreed that it would be valuable to enroll in Huiliao Insurance, while 135 (12%) disagreed with the statement, and 454 (40%) held a neutral opinion. The average score of perceptions of health risk was 4.13 (Table 2).

**Table 2.** Descriptive statistics.

Variable	Measurement	*N*	%
Area of residence	0 = Rural	333	29.3
	1 = Urban	803	70.7
Gender	0 = Male	552	48.6
	1 = Female	584	51.4
Age			
18–34	1 = if age was in this range, 0 = if not	238	21.0
35–59	1 = if age was in this range, 0 = if not	579	51.0
60–75	1 = if age was in this range, 0 = if not	319	28.1
Education status	0 = Not received college or above education	904	79.6
	1 = Received college or above education	232	20.4
Per capita monthly income	0 = Below or equal to average per capita income	788	69.4
	1 = Above average per capita income	348	30.6
Has at some stage bought private health insurance	0 = respondent has never bought private health insurance	605	53.3
	1 = respondent has previously bought private health insurance	531	46.7
Respondent or his/her family member has at some stage suffered from catastrophic diseases	0 = respondent or his/her family member did not suffer from catastrophic diseases	943	83.01
	1 = respondent or his/her family member ever suffered from catastrophic diseases	193	16.99
Average score on indicators	Measurement	Mean	SD
Awareness of Huiliao Insurance	Scale 1-5	3.29	0.79
Perceptions of its value	Scale 1-5	3.61	0.81
Health risk perceptions	Scale 1-5	4.13	0.55

We used Cronbach’s alpha to test the internal consistency of our scales. The Cronbach's alpha coefficients for the latent variables of health risk perceptions, awareness of Huimin Insurance, and perceptions of its value were 0.753, 0.778, and 0.805, respectively, all of which exceeded 0.7. We applied CFA to examine the validity of our psychological constructs. The CFA model demonstrated good fit with the root mean square error of approximation being 0.023, the Bentler comparative fit index being 0.993, and the standardized root mean square residual being 0.022. The validity of the constructs was confirmed since all their standardized factor loadings were significant with values being equal to or above 0.6.

### Hybrid choice model estimation results

Table 3 illustrates the findings from the HCM’s latent variable component, which encompasses both the structural and measurement models. The results of the structural model uncovered that higher health risk perceptions were associated with higher age (*β* = 0.263, *P* = .003; *β* = 0.358, *P* < .001), respondent or his/her family member ever suffering from catastrophic illness events (*β* = 0.219, *P* = .041), and respondents ever buying private health insurance (*β* = 0.126, *P* = .057). We found that awareness of Huiliao Insurance was significantly higher for those with higher health risk perceptions (*β* = 0.091, *P* = .056), a younger age (*β* = −0.292, *P* = .001; *β* = −0.665, *P* < .001) and for those who at some stage bought private health insurance (*β* = 1.048, *P* < .001). We also demonstrated that positive perceptions of Huiliao Insurance’s value were associated with rural respondents (*β* = −0.125, *P* = .074), higher health risk perceptions (*β* = 0.190, *P* < .001), and higher awareness of Huiliao Insurance (*β* = 0.528, *P* < .001). The results of the measurement model confirmed that each indicator had a strong relationship with its corresponding latent variable, with these relationships all being statistically significant.

**Table 3.** Results from HCM’s latent variable component.

	Health risk perceptions		Awareness of Huiliao insurance		Perceptions of its value	
	*β*	*P* ^a^	*β*	*P* ^a^	*β*	*P* ^a^
**Structural model**						
Gender	−0.086	0.145	0.088	0.120	−0.035	0.321
Age groups (ref: 20 ≤ age < 40)						
40 ≤ age < 60	0.263***	0.003	−0.292**	0.001	−0.073	0.202
age ≥ 60	0.358***	<0.001	−0.665***	*P* < 0.001	0.113	0.146
Residence	0.106	0.123	0.041	0.321	−0.125*	0.074
Respondent or his/her family member has at some stage suffered from catastrophic diseases	0.219**	0.041	−0.104	0.171	0.113	0.159
Has at some stage bought private health insurance	0.126*	0.057	1.048***	*P* < 0.001	−0.063	0.240
Health risk perceptions			0.091*	0.056	0.190***	*P* < 0.001
Awareness of Huiliao Insurance					0.528***	*P* < 0.001
**Measurement model**						
It is difficult to keep your body healthy all the time and not suffer from catastrophic diseases.	1.629***	<0.001				
Risks to your health are everywhere.	1.301***	<0.001				
More and more people suffer from catastrophic diseases. I am very worried about my health.	1.566***	<0.001				
Due to environmental pollution, food safety issues, and increased life pressure, the possibility of suffering from catastrophic diseases is increasing.	1.313***	<0.001				
Once someone becomes seriously ill, it will bring unbearable medical expenses to the family.	1.306***	<0.001				
I know Huiliao Insurance.			4.227***	<0.001		
I think Huiliao Insurance is a supplementary insurance to basic social health insurance schemes.			1.287***	<0.001		
I think Huiliao Insurance is an inclusive private health insurance.			1.546***	<0.001		
I think it is valuable to enroll in Huiliao Insurance.					2.665***	<0.001
I think Huiliao Insurance can reduce the financial burden of catastrophic diseases.					1.975***	<0.001
I am satisfied with Huiliao Insurance.					1.882***	<0.001

Significant at ***1%, **5%, *10%.

^a^We used robust standard errors to calculate *P*-values. If we had used normal standard errors, we would have more variables with significant *P*-values.

We also used the R package to run the DCE model with main effects to check whether people displayed preference heterogeneity. We found that all attributes and their levels had significant SDs, except for the moderate government involvement, which confirmed the existence of preference heterogeneity. Thus, we needed further models, such as the HCM, to systematically explore drivers of preference heterogeneity.

Table 4 shows the results from HCM’s choice model component. These results were important in understanding preference and preference heterogeneity of Huiliao Insurance. After considering preference heterogeneity from psychological constructs and socioeconomic and demographic factors, the SD of the expanded package covering convenience in medical treatment (*β* = −0.246, *P* = .174) became insignificant. And all the means of the attribute levels had the expected signs. Specifically, an increase in individual premium and deductibles was negatively associated with respondents’ utility (*β* = −0.007, *P* < .001; *β* = −0.036, *P* < .001). Conversely, a higher reimbursement ratio positively impacted utility (*β* = 0.049, *P* < .001). In terms of government involvement, both moderate and strong government involvement improved respondents’ utility compared to pure commercial operation (*β* = 0.764, *P* < .001; *β* = 0.880, *P* < .001). When comparing the basic package to the expanded value-added services, the latter consistently offered higher utility (*β* = 0.604, *P* < .001; *β* = 0.742, *P* < .001; *β* = 0.899, *P* < .001). We also found that the coefficients of interactions between awareness of Huiliao Insurance and ASC had positive signs (*β* = 0.170, *P* = .068), which meant that people with higher awareness of Huiliao Insurance derived higher disutility from the opt-out.

**Table 4.** Results from HCM’s choice model component.

	Coefficient		SD	
Attribute and attribute levels	*β*	*P* ^a^	*β*	*P* ^a^
Individual premiums	−0.007***	<.001	0.010***	<.001
Government involvement				
Purely commercially operated	ref			
Moderate government involvement	0.764***	<.001	−0.019	.444
Strong government involvement	0.880***	<.001	−0.641***	<.001
Deductibles	−0.036*	.060	0.110***	<.001
Reimbursement rates	0.049***	<.001	−0.03***	<.001
Value-added services				
Basic	ref			
Expanded 1	0.604***	<.001	−0.328*	.052
Expanded 2	0.742***	<.001	−0.848***	<.001
Expanded 3	0.899***	<.001	−0.246	.174
Opt-out	−3.889***	<.001		
Health risk perceptions*Opt-out	0.125	.131		
Awareness of Huiliao Insurance*Opt-out	0.170*	.068		
Perceptions of its value*opt-out	−0.004	.487		
Significant interaction terms ^b^				
Health risk perceptions*strong government involvement	−0.178**	.008		
Health risk perceptions*deductibles	−0.027**	.022		
Health risk perceptions*reimbursement rates	0.008**	.002		
Health risk perceptions*Expanded 1	0.254**	.002		
Health risk perceptions*Expanded 2	0.137*	.055		
Awareness of Huiliao Insurance*premiums	0.003**	.004		
Awareness of Huiliao Insurance*reimbursement rates	−0.005*	.051		
Awareness of Huiliao Insurance*Expanded 1	0.210**	.011		
Perceptions of its value*Expanded 1	−0.200**	.007		
Gender*deductibles	0.032**	.021		
Gender*reimbursement rates	−0.02***	<.001		
Residence*premiums	−0.004**	.004		
Residence*strong government involvement	0.210**	.038		
Residence*deductibles	−0.027*	.062		
Residence*Expanded 3	−0.419**	.005		
40 ≤ age < 60*reimbursement rates	−0.009**	.019		
age ≥ 60*reimbursement rates	−0.007*	.063		
Respondent or his/her family member has at some stage suffered from catastrophic diseases*premiums	0.003**	.035		
Respondent or his/her family member has at some stage suffered from catastrophic diseases*reimbursement rates	−0.006*	.095		
Has at some stage bought private insurance*reimbursement rates	0.006*	.070		
Has at some stage bought private insurance*Expanded 1	0.271**	.041		

Significant at ***1%, **5%, *10%.

^a^We used robust standard errors to calculate *P*-values. If we had used normal standard errors, we would have more interaction terms with significant *P*-values.

^b^Seven attribute levels in the main effects mixed logit model showed significant SDs and nine variables, including latent variables and socio-economic factors, needed to be interacted with attribute levels. Then totally, there were 63 interaction terms. And among these interaction terms, 21 (about 33.3%) were statistically significant and hence included in Table 4. The remaining insignificant interaction terms were left out.

Table 4 also displays the coefficients of interactions between psychological constructs and the attributes. We identified that people who agreed more with the statements on health risk perceptions preferred lower deductibles (*β* = −0.027, *P* = .022), higher reimbursement rates (*β* = 0.008, *P* = .002), and the first two expanded value-added services (*β* = 0.254, *P* = .002; *β* = 0.137, *P* = .055). While people with higher health risk perceptions showed lower preferences toward government strong involvement (*β* = −0.178, *P* = .008). We also found that respondents who agreed more with the three statements on awareness of Huiliao Insurance had less dislike toward the increase of premiums (*β* = 0.003, *P* = .004) and the decrease of reimbursement rates (*β* = −0.005, *P* = .051). And respondents with higher awareness of Huiliao Insurance showed higher preference toward Huiliao Insurance with the expanded package covering prevention and screening services (*β* = 0.210, *P* = .011). With regard to parameters of perceptions of Huiliao Insurance’s value, our findings indicate that people who had negative perceptions of its value showed higher preferences for the expanded package covering prevention and screening services (*β* = −0.200, *P* = .007).

Table 4 further illustrates the coefficients of interactions between socioeconomic and demographic factors and the attributes. We found that compared with men, women showed less dislike toward the increase of deductibles (*β* = 0.032, *P* = .021) and lower preferences toward the increase of reimbursement rates (*β* = −0.02, *P* < .001). Compared with rural residents, urban people derived higher disutility from the increase of premiums (*β* = −0.004, *P* = .004) and deductibles (*β* = −0.027, *P* = .062) and had lower preferences for the expanded package covering convenience in medical treatment (*β* = −0.419, *P* = .005). In contrast, urban residents expressed a more marked preference for strong government involvement (*β* = 0.210, *P* = .038). Compared with those aged 20–39, those with higher age showed lower preferences toward the increase of reimbursement rates (*β* = −0.009, *P* = .019; *β* = −0.007, *P* = .063). Compared with those without an experience of catastrophic illness events, those with such experience showed less dislike toward the increase of premiums (*β* = 0.003, *P* = .035) and lower preferences toward the increase of reimbursement rates (*β* = −0.006, *P* = .095). Compared with those who never bought any private health insurance, those who had at some stage bought insurance demonstrated higher preferences for Huiliao Insurance with higher reimbursement rates (*β* = 0.006, *P* = .070) and the expanded package covering prevention and screening services (*β* = 0.271, *P* = .041).

## Discussion

This study makes an important contribution to the literature as one of the very first studies that incorporates insurance attributes, socioeconomic and demographic status, and psychological factors when examining preferences for an insurance product. In line with expected utility theory and previous studies (Abiiro *et al.* 2014, Obse *et al.* 2016, Pendzialek *et al.* 2017), our study revealed that higher demand for such an insurance scheme was associated with lower premiums and deductibles, higher reimbursement rates, and the inclusion of more value-added services (such as prevention and screening services, disease diagnosis and treatment services, and convenience in medical treatment). We also found that more government involvement in the product design, publicity, and operation was associated with an increased demand for Huiliao Insurance. Moreover, our findings further indicated that psychological factors, such as health risk perceptions, knowledge of Huiliao Insurance, and perceptions of its value, as well as socioeconomic and demographic characteristics, were sources of heterogeneity in preferences for Huiliao Insurance.

Our findings confirmed our hypothesis that people who perceived higher health risks were more likely to have higher awareness of Huiliao Insurance and hold more positive perceptions of its value; in turn, people with better knowledge of its nature, i.e. an inclusive and complementary private health insurance, were also more likely to report more positive perceptions of its value. Health insurance is a risk management tool. Perceiving oneself to be at risk of potentially falling ill represents the first step in the path toward purchasing insurance (Kunreuther and Pauly 2006). Only once a person understands the characteristics of the Huiliao Insurance, can they start to appreciate its value (Gao *et al.* 2022). These results suggest that enhancing individuals’ perceptions of severe illness risk and their basic understanding of complementary inclusive health insurance can increase their perceptions of its value. Additionally, the associations we observed between older age and higher health risk perceptions and lower awareness of Huiliao Insurance are consistent with our conceptual understanding of aging in the Chinese context. Older adults are more susceptible to diseases and thus face higher health risk (Tingvold *et al.* 2022). Yet, they are more likely to have limited access to health information, especially online health information (Mubarak and Suomi 2022), whereas the internet is one of the most important promotion channels for Huimin Insurance (China Re Life 2023). Meanwhile, our findings suggest that those who at some stage bought private health insurance were more likely to have higher health risk perceptions and to have awareness of Huiliao Insurance, but were not more likely to show more positive recognition of its value. This subgroup of people is likely to experience high health risk and at the same time high levels of health insurance literacy, yet they do not value Huiliao Insurance, possibly due to overlapping coverage benefits between schemes. Further, respondents with an experience of catastrophic illness events were more likely to perceive higher health risk. The findings could be explained by a type of cognitive bias, i.e. availability heuristics, which is a mental shortcut to judge probabilities based on the easiness of examples or instances, and to recall the probability of the risk of catastrophic illness events (Kunreuther and Pauly 2006, Baicker *et al.* 2012). Additionally, the results showing that rural respondents were more likely to perceive the value of Huiliao Insurance suggest its market potential in rural areas.

Looking at preference heterogeneity from a psychological standpoint, we recognize that people perceiving themselves to be at greater health risk displayed a more marked preference for larger benefits (i.e. higher reimbursement rates, lower deductibles, and the expanded packages covering the early detection of physical health problems and related disease diagnosis and treatment services). Compared with those having lower health risk perceptions, those perceiving higher health risk usually tend to take up more risk management measures (Ferrer and Klein 2015), and thus regard Huiliao Insurance to have more attributes that can better protect them from catastrophic diseases. Meanwhile, our findings highlighting that people with higher health risk perceptions displayed a lower preference for a stronger government involvement can be explained by the fact that this group of people does not rely on government endorsement to increase trust in Huimin Insurance. Chinese people have high levels of trust in government (Statista Research Department 2025). By becoming actively involved in the design and promotion of the product, the Chinese government encouraged the development of Huimin Insurance (Hu *et al.* 2023) in a setting with a lower private insurance penetration rate than what was observed in other OECD countries (Wang *et al.* 2021a). Thus, compared with those with lower health risk perceptions, those having higher health risk perceptions rely less on strong government involvement to increase their perceived value of Huiliao Insurance, since they already have high demand for health insurance.

Additionally, in accordance with prior research (Panda *et al.* 2015, Gao *et al.* 2022), we found that knowing the nature and characteristics of Huiliao Insurance increased the probability of purchasing it. We also identified that those with higher awareness of Huiliao Insurance showed less dislike toward the increase of premiums and the decrease of reimbursement rates. People who have awareness of Huiliao Insurance well understand that it is inclusive and complementary to basic social health insurance, yet different from general private health insurance. They therefore display higher interest in Huiliao Insurance having higher premiums, but poorer benefits (i.e. lower reimbursement rates). The willingness of informed respondents to tolerate higher premiums and lower reimbursement rates suggests that, with the right communication and service structure, financially sustainable supplementary insurance models are feasible. This opens policy space for more contributory, actuarially sound complementary inclusive health insurance schemes that do not rely exclusively on public subsidies.

We then found that those who had higher awareness of Huiliao Insurance and negative perceptions of its value showed higher preferences toward the expanded package covering prevention and screening services. Such services attract those who know its nature and characteristics because these services are most relevant to the positioning of Huiliao Insurance in China’s multi-level health security system. Such services also spark high interest in Huiliao Insurance among those who have lower recognition of its value, suggesting that the expansion of these services could be used as an effective way to attract people, even those who have lower positive opinions on its value. Our findings thus indicate that strengthening the promotion of Huimin Insurance, enhancing public understanding of its characteristic and function, and enabling people to correctly estimate their risk of catastrophic illness events, especially when it is difficult to increase governmental involvement, are effective ways to increase people’s demand for such kind of health insurance. Therefore, governments and insurance providers should adopt targeted and segmented behavioral communication strategies to enhance residents’ understanding of severe illness risks and the role of Huimin Insurance. Psychological drivers such as health risk perception, insurance knowledge, and perceived scheme value should guide communication efforts. Online platforms, community health workers, and credible public endorsements can be leveraged to effectively reach high-risk yet underinformed groups. By improving insurance literacy and reshaping public perceptions through tailored outreach, participation in complementary inclusive health insurance can be fundamentally increased. Moreover, considering that even those who did not recognize the value of Huimin Insurance preferred the expanded value-added package covering prevention and screening services, we further suggest that such packages should rank first for the expansion of its value-added services.

In our study, preferences for Huimin Insurance appear to be more pronounced among population groups that generally lack access to other insurance products, such as women and rural residents. This is not surprising given that in the absence of other private or public products (Choi *et al.* 2018, Zhou *et al.* 2021), people are left to their own devices and end up incurring very high out-of-pocket expenditure (Fu 2022). It is unsurprising that the rural population also had higher preferences toward the expanded package covering convenience in medical treatment, since they have poor access to high-quality medical facilities and experts (Chen and Liu 2023). Given that rural and female residents exhibited a notable insensitivity to increased premiums and deductibles, we suggest that measures to enhance demand for complementary inclusive health insurance among these groups should receive more attention. Furthermore, if the goal is to expand the rural market for complementary inclusive health insurance, the inclusion of value-added services that enhance convenience in medical treatment should be carefully considered.

This study has the following limitations. First, even though some interaction terms were significant and the SD of the expanded three value-added services became insignificant in our HCM results, we are aware that there probably exist unobserved variables, either psychological constructs or socioeconomic and demographic factors, that might be sources of preference heterogeneity for Huiliao Insurance. We chose the three psychological factors mainly based on the sequential model of insurance choice (Kunreuther 1978). However, we recognize the existence of other psychological constructs that might drive preference heterogeneity for Huiliao Insurance. Moreover, while we applied Kunreuther's theoretical model (1978) to examine the relationship between perceptions of health risk, awareness of Huimin Insurance, and perceptions of its value, we cannot rule out the possibility that alternative theories may apply and may also be relevant to examine the relationship between these three constructs. Second, our study was based on a stated preference method. We could not rule out the possibility that one might behave differently in hypothetical and real scenarios. Third, our study relied on quota sampling to recruit participants from the sample database of KuRundata. While this method ensured a diverse sample, it is not without limitations. Specifically, it might have introduced potential sampling bias since our sample is not a representative sample of the population of the 13 cities in Liaoning. Given these limitations, future research should consider using alternative sampling methods and different samples to replicate our study and further validate our findings.

## Conclusions

Our paper also makes an important theoretical contribution by being one of the first studies to systematically integrate psychological constructs into insurance purchase decisions. In practical terms, our findings offer valuable insights for other countries grappling with similar challenges related to catastrophic health expenditures, even in the presence of basic social protection structures. Many LMICs are still suffering from catastrophic health expenditure, due to overstretched public health financing. Although most LMICs do not rely on financing arrangements like China’s Huimin Insurance, our study’s findings can still provide useful guidance to countries such as Brazil, South Africa, and Chile, where more than 16% of the population enjoys financial protection through the purchase of a private insurance scheme (Brazilian Institute of Geography and Statistics (IBGE) 2019, Ministry of Health 2022, Council for Medical Schemes 2023). While the specific features of these schemes may differ across countries, the underlying need to purchase coverage due to a void in public provision is essentially the same. As such, understanding how psychological factors shape preferences can be useful for other settings as well. To this effect, our work is specifically beneficial to guide the design and promotion of appropriate health protection measures against illness, especially catastrophic illnesses, and has potential applications in long-term care.

## Supplementary Material

czaf056_Supplementary_Data

## Data Availability

The data of this study are available from the first author, W.Q., upon request.

## Reflexivity statement

The authors include three females and two males and span multiple levels of seniority. Three members specialize in health policy, while the other two bring expertise in psychology and computer engineering, respectively. All authors have extensive experience conducting quantitative research in low- and middle-income countries, with a strong focus on China.
