# Supplementary material for: Psychological constructs and preferences for a complementary inclusive health insurance: a hybrid choice model
Source: Health Policy Plan. 2025 Aug 21;40(9):981–91. doi: 10.1093/heapol/czaf056 (PMC12516031; doi:10.1093/heapol/czaf056)
Supplement: czaf056_Supplementary_Data [file czaf056_supplementary_data.zip › Appendix 1.docx]

Appendix 1 The Formal DCE Questionnaire (Block 1)

[Huiliao Insurance] is a commercial health insurance launched under the guidance of Liaoning Province Health Security Bureau in conjunction with 14 insurance companies, including PICC Property and Casualty Company Limited (PICC P&C) and China Pacific Insurance (Group) Co., Ltd. (CPIC), to alleviate the burden of catastrophic disease costs on the public. Compared to other private health insurance options, Huiliao Insurance offers low premiums, no age limit, and no health condition limit, making it available to all residents of Liaoning (excluding Dalian) who have basic social health insurance coverage.

**Section I:**

1. Based on your knowledge of Huiliao Insurance, please place a "√" in the number corresponding to your selected option.

| Statement | Strongly Disagree | Disagree | Neutral | Agree | Strongly Agree |
| --- | --- | --- | --- | --- | --- |
| I know Huiliao Insurance. | 1 | 2 | 3 | 4 | 5 |
| I think Huiliao Insurance is a complementary insurance to basic social health insurance schemes. | 1 | 2 | 3 | 4 | 5 |
| I think Huiliao Insurance is an inclusive private health insurance. | 1 | 2 | 3 | 4 | 5 |
| I think it is valuable to enroll in Huiliao Insurance. | 1 | 2 | 3 | 4 | 5 |
| I think Huiliao Insurance can reduce the financial burden of catastrophic diseases. | 1 | 2 | 3 | 4 | 5 |
| I am satisfied with Huiliao Insurance. | 1 | 2 | 3 | 4 | 5 |

2. Based on your actual views, please place a “√” in the number corresponding to your selected option.

| Statement | Strongly Disagree | Disagree | Neutral | Agree | Strongly  Agree |
| --- | --- | --- | --- | --- | --- |
| It is difficult to keep your body healthy all the time and not suffer from catastrophic diseases. | 1 | 2 | 3 | 4 | 5 |
| Risks to your health are everywhere. | 1 | 2 | 3 | 4 | 5 |
| More and more people suffer from catastrophic diseases. I am very worried about my health. | 1 | 2 | 3 | 4 | 5 |
| Due to environmental pollution, food safety issues, and increased life pressure, the possibility of suffering from catastrophic diseases is increasing. | 1 | 2 | 3 | 4 | 5 |
| Once someone becomes seriously ill, it will bring unbearable medical expenses to the family. | 1 | 2 | 3 | 4 | 5 |

**Section II: Preference Survey**

We have designed Huiliao Insurance as a product with five core attributes (government involvement, individual premiums, deductibles of benefits within and outside the social health insurance catalog, reimbursement rates, and value-added services).

Deductibles refer to a specified threshold amount stipulated in the insurance policy, below which the insured bears the losses entirely and the insurer assumes no liability for payment. In other words, it represents out-of-pocket expenses that do not meet the reimbursement criteria.

Next, you will see 7 different groups of Huiliao Insurance options. Please check the Huiliao Insurance option you prefer based on your actual needs.

Choice set 1

|  | **Huiliao Insurance 1** | **Huiliao Insurance 2** |
| --- | --- | --- |
| **Individual premiums** | **130 RMB/**year | **210 RMB/**year |
| **Government involvement** | **Government strong involvement**  (Deeply involved in product operation, design, and publicity) | **Purely commercially operated**  (Largely uninvolved in its operation) |
| **Deductibles of benefits within and outside the health insurance catalog** | **18,000 RMB**  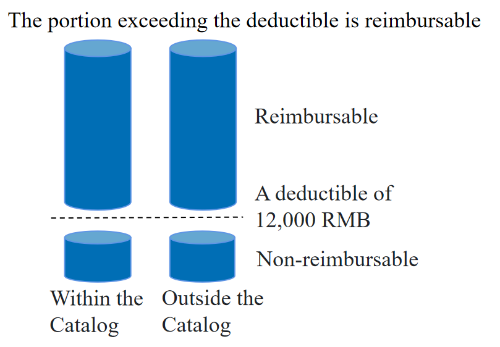 | **12,000 RMB**  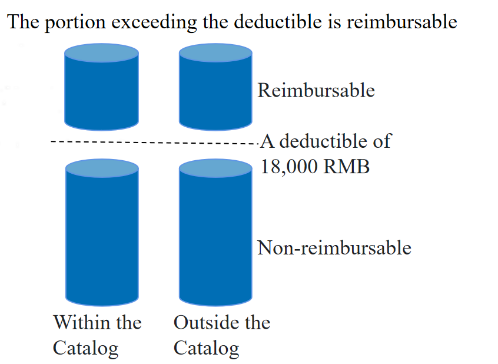 |
| **Reimbursement rates** | **50%** | **80%** |
| **Value-added services** | **Discounts on drug purchases** | **Discounts on drug purchases + prevention and screening services**  (Early screening for major diseases, discounts on physical examinations, etc.) |

**Which do you prefer？**

**A. Huiliao insurance 1 B. Huiliao insurance 2 C. None of the above options**

Choice set 2

|  | **Huiliao Insurance 1** | **Huiliao Insurance 2** |
| --- | --- | --- |
| **Individual premiums** | **210 RMB/**year | **130 RMB/**year |
| **Government involvement** | **Government strong involvement**  (Deeply involved in product operation, design, and publicity) | **Government moderate involvement**  (Moderately involved product operation, design, and publicity) |
| **Deductibles of benefits within and outside the health insurance catalog** | **12,000 RMB**  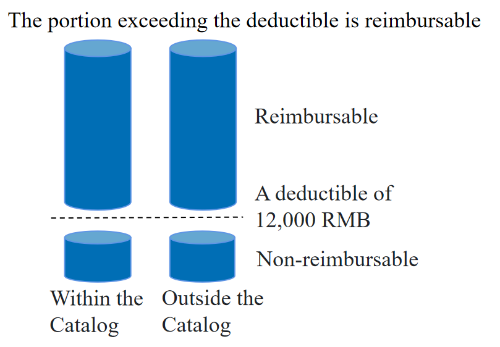 | **18,000 RMB**  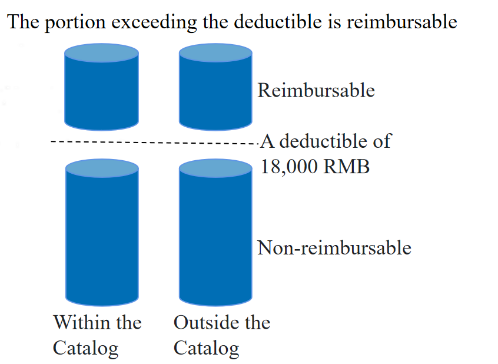 |
| **Reimbursement rates** | **65%** | **65%** |
| **Value-added services** | **Discounts on drug purchases + disease diagnosis and treatment services**  (video medical inquiries, traditional Chinese medicine diagnosis and treatment, etc.) | **Discounts on drug purchases + ​​medical green channel services**  (Green channel of outpatient service, medical guidance, etc.) |

**Which do you prefer？**

**A. Huiliao insurance 1 B. Huiliao insurance 2 C. None of the above options**

Choice set 3

|  | **Huiliao Insurance 1** | **Huiliao Insurance 2** |
| --- | --- | --- |
| **Individual premiums** | **210 RMB/**year | **130 RMB/**year |
| **Government involvement** | **Government moderate involvement**  (Moderately involved product operation, design, and publicity) | **Government strong involvement**  (Deeply involved in product operation, design, and publicity) |
| **Deductibles of benefits within and outside the health insurance catalog** | **15,000 RMB**  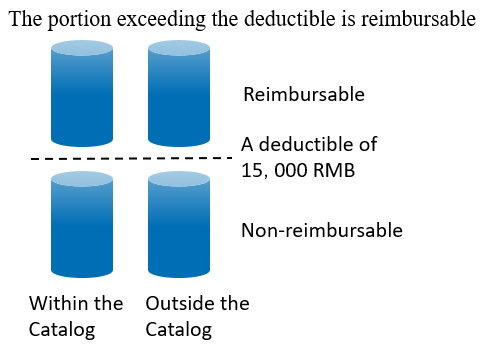 | **15,000 RMB**  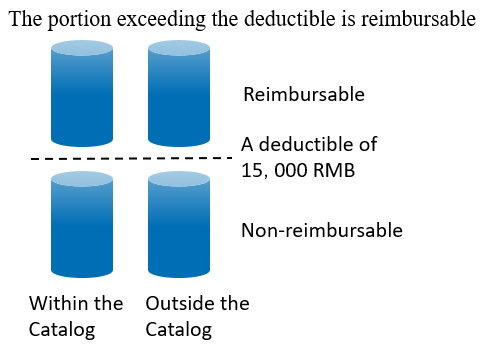 |
| **Reimbursement rates** | **50%** | **80%** |
| **Value-added services** | **Discounts on drug purchases + prevention and screening services**  (Early screening for major diseases, discounts on physical examinations, etc.) | **Discounts on drug purchases + medical green channel services**  (Green channel of outpatient service, medical guidance, etc.) |

**Which do you prefer？**

**A. Huiliao insurance 1 B. Huiliao insurance 2 C. None of the above options**

Choice set 4

|  | **Huiliao Insurance 1** | **Huiliao Insurance 2** |
| --- | --- | --- |
| **Individual premiums** | **130 RMB/**year | **210 RMB/**year |
| **Government involvement** | **Government strong involvement**  (Deeply involved in product operation, design, and publicity) | **Government moderate involvement**  (Moderately involved product operation, design, and publicity) |
| **Deductibles of benefits within and outside the health insurance catalog** | **15,000 RMB**  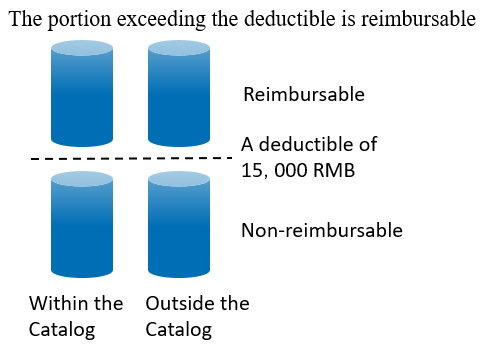 | **15,000 RMB**  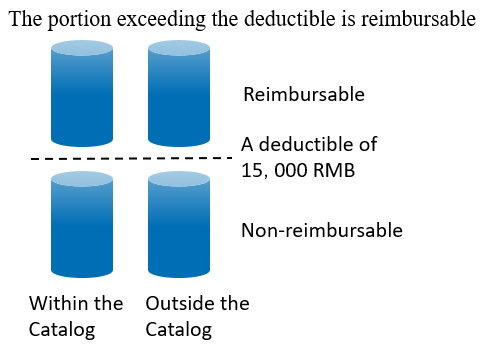 |
| **Reimbursement rates** | **80%** | **50%** |
| **Value-added services** | **Discounts on drug purchases + medical green channel services**  (Green channel of outpatient service, medical guidance, etc.) | **Discounts on drug purchases + disease diagnosis and treatment services**  (video medical inquiries, traditional Chinese medicine diagnosis and treatment, etc.) |

**Which do you prefer？**

**A. Huiliao insurance 1 B. Huiliao insurance 2 C. None of the above options**

Choice set 5

|  | **Huiliao Insurance 1** | **Huiliao Insurance 2** |
| --- | --- | --- |
| **Individual premiums** | **130 RMB/**year | **210 RMB/**year |
| **Government involvement** | **Government moderate involvement**  (Moderately involved product operation, design, and publicity) | **Purely commercially operated**  (Largely uninvolved in its operation) |
| **Deductibles of benefits within and outside the health insurance catalog** | **18,000 RMB**  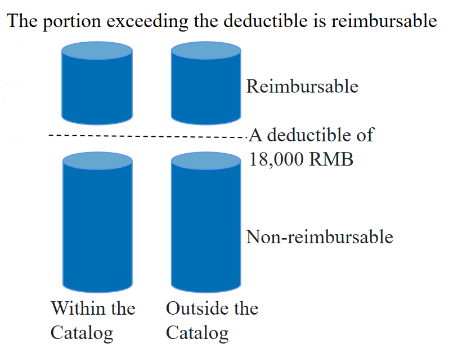 | **12,000 RMB**  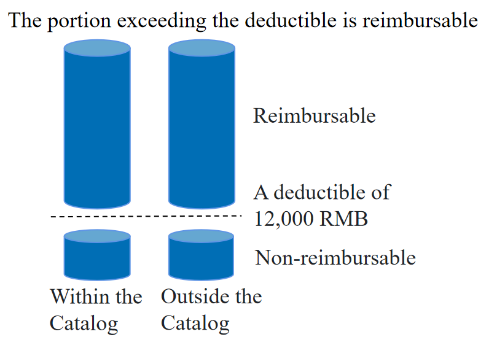 |
| **Reimbursement rates** | **50%** | **80%** |
| **Value-added services** | **Discounts on drug purchases + disease diagnosis and treatment services**  (video medical inquiries, traditional Chinese medicine diagnosis and treatment, etc.) | **Discounts on drug purchases + prevention and screening services**  (Early screening for major diseases, discounts on physical examinations, etc.) |

**Which do you prefer？**

**A. Huiliao insurance 1 B. Huiliao insurance 2 C. None of the above options**

Choice set 6

|  | **Huiliao Insurance 1** | **Huiliao Insurance 2** |
| --- | --- | --- |
| **Individual premiums** | **170 RMB/**year | **170 RMB/**year |
| **Government involvement** | **Purely commercially operated**  (Largely uninvolved in its operation) | **Government moderate involvement**  (Moderately involved product operation, design, and publicity) |
| **Deductibles of benefits within and outside the health insurance catalog** | **15,000 RMB**  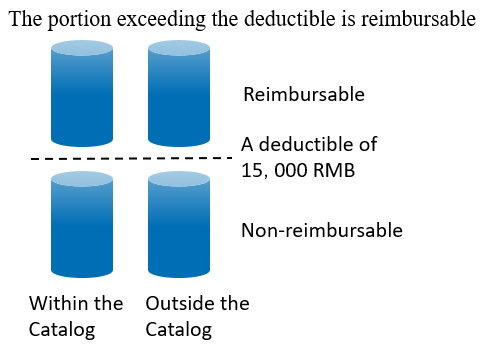 | **15,000 RMB**  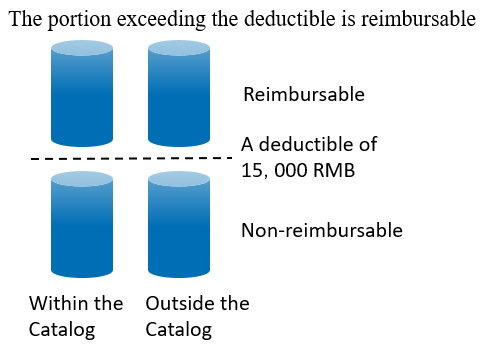 |
| **Reimbursement rates** | **80%** | **50%** |
| **Value-added services** | **Discounts on drug purchases + medical green channel services**  (Green channel of outpatient service, medical guidance, etc.) | **Discounts on drug purchases** |

**Which do you prefer？**

**A. Huiliao insurance 1 B. Huiliao insurance 2 C. None of the above options**

Choice set 7

|  | **Huiliao Insurance 1** | **Huiliao Insurance 2** |
| --- | --- | --- |
| **Individual premiums** | **210 RMB/**year | **130 RMB/**year |
| **Government involvement** | **Purely commercially operated**  (Largely uninvolved in its operation) | **Government strong involvement**  (Deeply involved in product operation, design, and publicity) |
| **Deductibles of benefits within and outside the health insurance catalog** | **18,000 RMB**  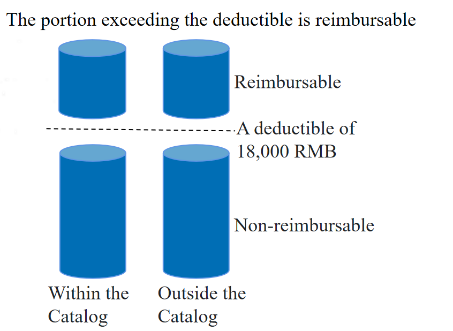 | **12,000 RMB**  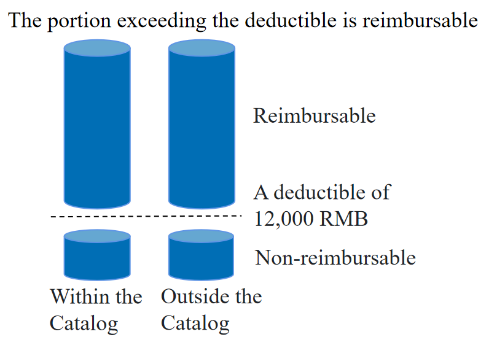 |
| **Reimbursement rates** | **50%** | **80%** |
| **Value-added services** | **Discounts on drug purchases** | **Discounts on drug purchases + prevention and screening services**  (Early screening for major diseases, discounts on physical examinations, etc.) |

Note: Deductibles applicable to both in-catalog and out-of-catalog services refer to the annual cumulative amount; identical reimbursement rates are provided for all covered services, regardless of their inclusion in the health insurance catalog.

**Which do you prefer？**

**A. Huiliao insurance 1 B. Huiliao insurance 2 C. None of the above options**

**Section III: Demographic Information**

1. If you have a sum of money to invest, which investment option would you prefer?

(1) High-risk, high-return projects
(2) Slightly higher risk, slightly higher return projects
(3) Moderate risk, moderate return projects
(4) Slightly lower risk, slightly lower return projects
(5) No willingness to assume any risk

2. Have you ever purchased any commercial health insurance?

(1) Yes (2) No

3. Before this survey, have you ever heard of or learned about Huiliao Insurance?

(1) Yes (2) No

(If the previous answer is "Yes", please proceed to this question.) What is the primary channel through which you obtained information about Huiliao Insurance?

(1) Government websites and related apps

(2) Recommendations from insurance company staff
(3) Traditional media such as television, radio, newspapers, etc.
(4) Social media platforms such as WeChat, Weibo, Douyin, etc.
(5) Information from family and friends

4. Have you ever purchased Huiliao Insurance?

(1) Yes (2) No

5. Have you ever filed a claim for Hui Liao Insurance?

(1) Yes (2) No

6. The new year's Huiliao Insurance will be launched in the coming months. Please place a “√” in the number corresponding to your selected option based on your actual thoughts:

| Statement | Strongly Disagree | Disagree | Neutral | Agree | Strongly Agree |
| --- | --- | --- | --- | --- | --- |
| I will purchase Huiliao Insurance for myself. | 1 | 2 | 3 | 4 | 5 |
| I will purchase Huiliao Insurance for my immediate family. | 1 | 2 | 3 | 4 | 5 |
| I will recommend Huiliao Insurance to others. | 1 | 2 | 3 | 4 | 5 |

7. Your gender:
(1) Male (2) Female

8. Your age (in years): ___ years

9. Your current place of residence:
(1) Shenyang (2) Anshan (3) Fushun (4) Benxi (5) Dandong (6) Jinzhou

(7) Yingkou (8) Fuxin (9) Liaoyang (10) Panjin (11) Tieling (12) Chaoyang

(13) Huludao (14) Dalian

10. Your Hukou type:
(1) Rural (2) Urban

11. Your type of residence:
(1) Rural (2) Urban

12. Your marital status:
(1) Single (2) Married (3) Divorced (4) Widowed

13. Your education status:
(1) No formal education (2) Primary school (3) Middle school
(4) High school/Technical school/Secondary vocational school
(5) College/University (6) Master's degree or higher

14. What is your main occupation?
(1) Civil servant or employee in a public institution (2) State-owned enterprise employee

(3) Private sector employee (4) Self-employed business owner (5) Retired

(6) Agricultural worker (7) Freelancer (8) Student

(9) Unemployed in urban and rural areas (10) Other

15. How many people are there in your household (including all members who have lived together in the past six months, excluding domestic workers)? ____ people.

16. What is your household's annual disposable income?
(1) RMB 30,000 or below (including RMB 30,000)

(2) RMB 30,000–80,000 (including RMB 80,000)

(3) RMB 80,000–130,000 (including RMB 130,000)

(4) RMB 130,000–180,000 (including RMB 180,000)

(5) RMB 180,000–230,000 (including RMB 230,000)

(6) RMB 230,000–280,000 (including RMB 280,000)

(7) RMB 280,000–300,000 (including RMB 300,000)

(8) Above RMB 300,000

17. Have you ever suffered from catastrophic diseases (such as malignant tumors, liver and kidney diseases, etc., which involve high treatment costs and severely affect the patient's and their family's normal work and life for an extended period)?
(1) Yes (2) No

18. Has any of your family members ever suffered from catastrophic diseases?
(1) Yes (2) No

19. How often do you undergo physical check-ups?
(1) Once or twice a year (2) Once every two years (3) Not had a check-up in over two years

20. How often do you exercise?
(1) More than twice a week (2) Once a week (3) Occasionally (4) Rarely

21. Do you actively seek information that is beneficial to your health?
(1) Never (2) Rarely (3) Unsure (4) Sometimes (5) Always

22. How would you rate your health status?
(1) Healthy (2) Average (3) Unhealthy

23. What is your annual medical expenditure?
(1) 2,000 yuan or less
(2) 2,000 - 10,000 yuan (including 10,000 yuan)
(3) 10,001 - 30,000 yuan (including 30,000 yuan)
(4) 30,001 - 100,000 yuan (including 100,000 yuan)
(5) Above 100,000 yuan

24. How many chronic diseases do you have?
(1) None (2) One (3) Two (4) Three or more

25. Which basic social health insurance do you currently participate in?
(1) Urban Employee Basic Medical Insurance
(2) Urban and Rural Resident Basic Medical Insurance
(3) None

26. Is the basic social health insurance you participate in within Liaoning Province or outside of it?
(1) Within Liaoning Province
(2) Outside Liaoning Province
(3) None
