# Supplementary material for: Psychological constructs and preferences for a complementary inclusive health insurance: a hybrid choice model
Source: Health Policy Plan. 2025 Aug 21;40(9):981–91. doi: 10.1093/heapol/czaf056 (PMC12516031; doi:10.1093/heapol/czaf056)
Supplement: czaf056_Supplementary_Data [file czaf056_supplementary_data.zip › Appendix 2.docx]

**Appendix 2 The Hybrid Choice Model in this Study**

Latent variable model

Psychological constructs, such as insurance perceptions and health risk perceptions, were influenced by socioeconomic and demographic factors. We used a Multiple Indicator Multiple Cause (MIMIC) model to identify the relationship between latent variables, socioeconomic and demographic factors, and psychological indicators. The MIMIC model includes the structural model and the measurement model (Anderson and Gerbing, 1988). In the structural model, the $l$-th latent variable $\alpha_{il}$ for individual $i$ is expressed as:

$$\alpha_{il}=\gamma_{l}^{'}Z_{i}+\eta_{il}$$

$I_{i,k}=\left\{ \begin{aligned} 1, &if \zeta_{k}\alpha_{il}<\tau_{k,1} \\ 2, &if \tau_{k,1}\leq\zeta_{k}\alpha_{il}<\tau_{k,2} \\ \vdots\\ s, &if \zeta_{k}\alpha_{il}\geq\tau_{k,s-1} \end{aligned} \right.$ (1)

where $Z_{i}$ represents socioeconomic and demographic factors, $\gamma_{l}$ captures the effect of socioeconomic and demographic factors on the latent variables, and $\eta_{il}$ is a normally distributed random disturbance. In the measurement model,$I_{ikl}$ represents the $k$-th psychological indicator for $l$-th latent variable and is explained by this latent variable. We applied an ordered logit specification for all psychological indicators (Arora *et al.*, 2022). The possibility of observing a value s for indicator $I$ can be expressed that:

$L(I_{i,l};\tau,\zeta,\alpha_{il})=\sum_{s=1}^{S} \delta_{(I_{i,k}=s)}[\frac{e^{\tau_{k,s}-\zeta_{k}\alpha_{il}}}{1+e^{\tau_{k,s}-\zeta_{k}\alpha_{il}}}-\frac{e^{\tau_{k,s-1}-\zeta_{k}\alpha_{il}}}{1+e^{\tau_{k,s-1}-\zeta_{k}\alpha_{il}}}]$ (2)

where $\zeta_{k}$ measures the effect of the latent variable $\alpha_{il}$ on indicator $I$, $\tau_{k,s}$ is a vector of threshold parameters for indicator $I$, and$\delta=1$ if $I_{i,k}=s$, otherwise $\delta=0$.

Choice model

For individual $i$ maximizing his/her utility, the utility of choosing alternative $j$ in choice set $t$ is expressed as a sum of a deterministic part $V_{ijt}$and a random part $\varepsilon_{ijt}$. And the deterministic part is a linearly additive function of preferences for Huiliao Insurance attributes and the insurance itself (versus no insurance) (Hensher *et al.*, 2015).

$U_{ijt}=V_{ijt}+\varepsilon_{ijt}=ASC+\beta^{'}X_{ijt}+\varepsilon_{ijt}$ (3)

where $\beta$ is a vector of preference estimates and $X_{ijt}$ is a vector of Huiliao Insurance attributes. The alternative specific constant (ASC) captures the utility of choosing the opt-out option.

Then, for individual $i$, the likelihood of choosing alternative $j$ choices conditional on $\beta$ and ASC can be represented as:

$L(C_{ijt};\beta,ASC)=\frac{exp(V_{ijt})}{\sum_{j=1}^{J} exp(V_{ijt})}$ (4)

Where $C_{ijt}$is set to 1 if individual $i$ chooses alternative $j$ in choice set $t$, and 0 otherwise.

Further, $\beta$ is often treated as not fixed, but with probability density $f(\beta|\Omega)$. The deviation of $\beta$ can also be introduced deterministically, which is modeled as a function of socioeconomic and demographic factors. Taking $\beta_{I}$, representing preference of premiums, as an example, we have that:

$\beta_{I}=\mu_{I}+ \omega^{'}Z_{i}+\theta_{I}$ (5)

Where $\mu_{I}$ is the mean, $\omega$ is a vector capturing preference heterogeneity from different socioeconomic and demographic factors, $Z_{i}$. And $\theta_{I}\sim N(0, \sigma_{\theta,I}^{2})$ captures random heterogeneity for attribute I.

In addition, as shown above, preference heterogeneity for Huiliao Insurance may also arise from psychological constructs. Thus, the preference of premiums,$\beta_{I}$, now becomes:

$\beta_{I}=\mu_{I}+ \omega^{'}Z_{i}+\nu^{'}{\alpha_{il}+\theta}_{I}$ (6)

where $\nu$ is a vector capturing the effects of latent psychological constructs on preferences.

To measure preference heterogeneity, interactions between socioeconomic and demographic factors, psychological constructs, and the attribute levels with significant standard deviations (SDs) (p<0.05) were added to the utility function (Hensher *et al.*, 2015). Additionally, latent variables could also affect the utility of Huiliao Insurance itself (versus no insurance). Thus, the utility function is expressed as:

$U_{ijt}=V_{ijt}+\varepsilon_{ijt}=ASC+\sum\theta_{l}ASC\times\alpha_{il}+\sum\mu_{ijt}X_{ijt}+\sum\omega_{ijt}Z_{i}X_{ijt}+\sum v_{ijt}\alpha_{il}X_{ijt}+\varepsilon_{ijt}$ (7)

where $\theta_{ln}$ represents the effects of latent variable $\alpha_{ln}$ interacting with the opt-out option. Then, the likelihood function of HCM is expressed as:

$LL(C_{ijt},I_{i,l};\tau,\zeta,\gamma,{\omega,\upsilon,\Omega}_{\beta})=\sum_{i=1}^{N} ln\int_{\beta} \int_{a} L\left( C_{ijt};\beta\right)\left( \prod_{k=1}^{K} L\left( I_{i,l};\tau,\zeta,\alpha_{il} \right) \right)f\left( \beta| \Omega\right)g\left( \alpha\right)d\alpha d\beta$ (8)

where K equals the sum of the number of indicators of all psychological constructs. In our study, we have K=3. We assumed all attributes and their levels with normal distributions and generated 800 draws using Modified Latin Hypercube Sampling (MLHS) algorithm to simulate the likelihood.

**References**

Anderson JC, Gerbing DW. 1988. Structural equation modeling in practice: A review and recommended two-step approach. *Psychological Bulletin* **103**: 411–23.

Arora N, Crastes dit Sourd R, Hanson K, Woldesenbet D, Seifu A, Quaife M. 2022. Linking health worker motivation with their stated job preferences: A hybrid choice analysis in Ethiopia. *Social Science & Medicine* **307**: 115151.
